# Human NREM Sleep Promotes Brain-Wide Vasomotor and Respiratory Pulsations

**DOI:** 10.1523/JNEUROSCI.0934-21.2022

**Published:** 2022-03-23

**Authors:** Heta Helakari, Vesa Korhonen, Sebastian C. Holst, Johanna Piispala, Mika Kallio, Tommi Väyrynen, Niko Huotari, Lauri Raitamaa, Johanna Tuunanen, Janne Kananen, Matti Järvelä, Timo Tuovinen, Ville Raatikainen, Viola Borchardt, Hannu Kinnunen, Maiken Nedergaard, Vesa Kiviniemi

**Affiliations:** ^1^Oulu Functional Neuroimaging, Department of Diagnostic Radiology, Oulu University Hospital, 90220 Oulu, Finland; ^2^Medical Imaging, Physics and Technology, Faculty of Medicine, University of Oulu, 90220 Oulu, Finland; ^3^Medical Research Center, Oulu University Hospital, 90220 Oulu, Finland; ^4^Neurobiology Research Unit, University of Copenhagen, 2200 Copenhagen, Denmark; ^5^Clinical Neurophysiology, Oulu University Hospital, 90220 Oulu, Finland; ^6^Oura Health, 90590 Oulu, Finland; ^7^Center of Translational Neuromedicine, University of Copenhagen, 2200 Copenhagen, Denmark; ^8^Center of Translational Neuromedicine, University of Rochester, Rochester, New York 14642

**Keywords:** brain pulsations, fast fMRI, glymphatic clearance, sleep, slow-wave EEG, spectral power

## Abstract

The physiological underpinnings of the necessity of sleep remain uncertain. Recent evidence suggests that sleep increases the convection of cerebrospinal fluid (CSF) and promotes the export of interstitial solutes, thus providing a framework to explain why all vertebrate species require sleep. Cardiovascular, respiratory and vasomotor brain pulsations have each been shown to drive CSF flow along perivascular spaces, yet it is unknown how such pulsations may change during sleep in humans. To investigate these pulsation phenomena in relation to sleep, we simultaneously recorded fast fMRI, magnetic resonance encephalography (MREG), and electroencephalography (EEG) signals in a group of healthy volunteers. We quantified sleep-related changes in the signal frequency distributions by spectral entropy analysis and calculated the strength of the physiological (vasomotor, respiratory, and cardiac) brain pulsations by power sum analysis in 15 subjects (age 26.5 ± 4.2 years, 6 females). Finally, we identified spatial similarities between EEG slow oscillation (0.2–2 Hz) power and MREG pulsations. Compared with wakefulness, nonrapid eye movement (NREM) sleep was characterized by reduced spectral entropy and increased brain pulsation intensity. These effects were most pronounced in posterior brain areas for very low-frequency (≤0.1 Hz) vasomotor pulsations but were also evident brain-wide for respiratory pulsations, and to a lesser extent for cardiac brain pulsations. There was increased EEG slow oscillation power in brain regions spatially overlapping with those showing sleep-related MREG pulsation changes. We suggest that reduced spectral entropy and enhanced pulsation intensity are characteristic of NREM sleep. With our findings of increased power of slow oscillation, the present results support the proposition that sleep promotes fluid transport in human brain.

**SIGNIFICANCE STATEMENT** We report that the spectral power of physiological brain pulsation mechanisms driven by vasomotor, respiration, and cardiac rhythms in human brain increase during sleep, extending previous observations of their association with glymphatic brain clearance during sleep in rodents. The magnitudes of increased pulsations follow the rank order of vasomotor greater than respiratory greater than cardiac pulsations, with correspondingly declining spatial extents. Spectral entropy, previously known as vigilance and as an anesthesia metric, decreased during NREM sleep compared with the awake state in very low and respiratory frequencies, indicating reduced signal complexity. An EEG slow oscillation power increase occurring in the early sleep phase (NREM 1–2) spatially overlapped with pulsation changes, indicating reciprocal mechanisms between those measures.

## Introduction

A notable feature of the mammalian brain is that it consists of almost 80% water (Tait et al., 2008), with a near absence of connective tissue. Also, the brain floats in cerebrospinal fluid (CSF) within the closed cavity of the skull. These physical characteristics enable brain-wide pulsations that drive fast fluid movement within the neuropil. In the early 20th century Hans Berger described three forms of brain pressure pulsations, “eine pulsatorische, eine respiratorische und vasomotorische Bewenung” (Berger, 1901); his studies of pulsatile brain activity lead the invention of electroencephalography (EEG).

Nearly a century later, the same triad of cardiovascular, respiratory, and slow, spontaneous vasomotor waves driven by arteries can be noninvasively imaged throughout the brain by magnetic resonance encephalography (MREG), which is a fast variant of functional magnetic resonance imaging (fMRI; Kiviniemi et al., 2016) designed to maximize temporal accuracy while maintaining adequate image quality. In addition to MREG, several fMRI imaging techniques have shown distinct cardiac, respiratory (Klose et al., 2000; Posse et al., 2013; Yamada et al., 2013; Dreha-Kulaczewski et al., 2015; Matsumae et al., 2019; Sloots et al., 2020), and very low-frequency (<0.1 Hz; Fultz et al., 2019) pulsations in the living human brain. The pulsations must bear some relation with the normal resting heart rate, which ranges between 1 and 1.67 Hz in humans (Mason et al., 2007) and respiratory rate, which is within or close to a range from 0.16 to 0.33 Hz (Russo et al., 2017); both of these rates decrease during nonrapid eye movement (NREM sleep; Penzel et al., 2003). Compared with other techniques, MREG enables robust detection and separation of physiological brain pulsations in the whole brain because of the greater spectral resolution in the range of 0–5 Hz, which is obtained without signal aliasing (Huotari et al., 2019; Raitamaa et al., 2019; Tuovinen et al., 2020).

Sleep is known to elicit electrohydrodynamic changes in brain tissue (Ding et al., 2016), which are driven by the physiological pulsations (Mestre et al., 2018). Also, prior reports in rodents showed that natural sleep and certain anesthetic regimens can induce a sharp increase in the influx of CSF to brain tissue, which further announces an increase in slow-wave electrophysiological activity (Xie et al., 2013; Hablitz et al., 2019). A recent study in mice associated 0.1 Hz vasomotor wave activity with increased paravascular solute transport (van Veluw et al., 2020), thus serving as an additional driver of fluid convection supplementing the previously presented cardiovascular brain pulsatility (Iliff et al., 2013; Mestre et al., 2018). In humans, NREM sleep and drowsiness are associated with high amplitude of very low-frequency fluctuations of blood oxygenation level-dependent (BOLD) signals (Fukunaga et al., 2006; Horovitz et al., 2008; Chang et al., 2016; Wong et al., 2016), which some suggest arise from vasomotion (Biswal et al., 1995; Kiviniemi et al., 2000; Silvani et al., 2004; Wang et al., 2008; Rayshubskiy et al., 2014). Furthermore, simultaneous EEG-fMRI study in humans linked increased delta wave amplitude during sleep with decreased cerebral blood volume followed by increased flow of CSF into the fourth ventricle (Fultz et al., 2019). Also, the previous human neuroimaging literature suggests that respiratory pulsation is the main pulsatory driver of CSF flow and intracerebral venous blood flow (Dreha-Kulaczewski et al., 2015; Matsumae et al., 2019; Vinje et al., 2019). Based on these various findings, we concluded that brain pulsations may affect the influx of CSF in a manner related to the state of consciousness and in association with slow-wave electrophysiological activity.

Based on this scenario, we used whole-brain MREG with synchronous EEG sleep monitoring during wakefulness and sleep to test the hypotheses that MREG pulsations change during sleep and that the pulsations occur in association with EEG slow oscillations. By inducing a T2* MRI signal, MREG allows us to study brain fluid dynamics originating not only from blood oxygenation (i.e., susceptibility related BOLD effect), but also the dynamics arising from the CSF space and interstitial fluid changes. The short repetition time (TR; 100 ms) and the used read pulse flip angle (FA; 5°) both increase inflow effects and sensitize detection of the steady-state precession alterations induced in the brain MRI data by relatively sudden cardiorespiratory impulses. We studied spectral entropy and the power sum of the MREG signal to assess sleep-related pulsation changes and demonstrated their relation to EEG slow oscillations (0.02–2 Hz) with spatial colocalization. Overall, we found significant changes in vasomotor, respiratory, and cardiac pulsations during NREM sleep compared with awake, mostly in brain areas overlapping with those showing EEG slow oscillation changes.

## Materials and Methods

### Subjects

**Figure 1.**
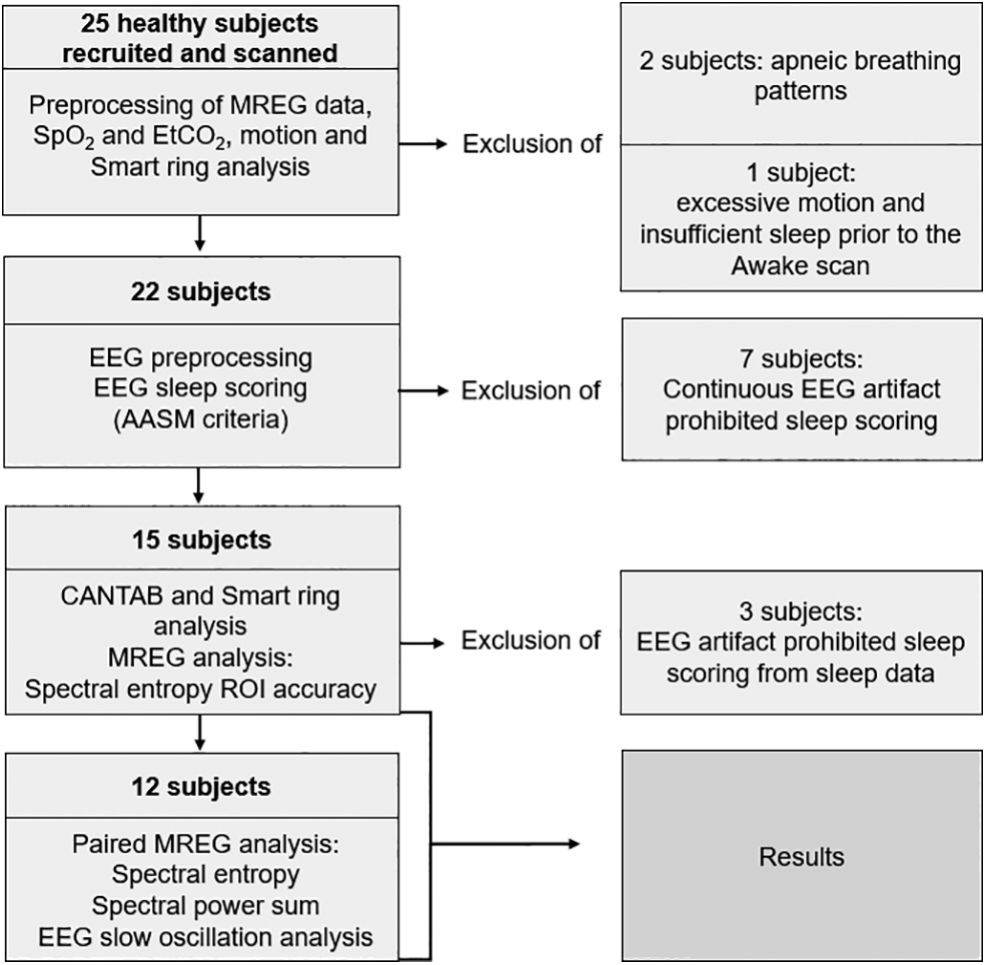
Data processing and exclusion of subjects. Twenty-five subjects were recruited and scanned in the study. The final analyses included 15 and 12 subjects, whose results are presented in the article. MREG, magnetic resonance encephalography; AASM, American Academy of Sleep Medicine; EtCO, end-tidal carbon dioxide; SpO, fingertip peripheral; ROI, region of interest.

Twenty-five subjects (age 28.0 ± 5.9 years, 11 females) participated in the study, which was approved by the Regional Ethics Committee of the Northern Ostrobothnia Hospital District. Written informed consent was obtained from all participants, according to requirements of the Declaration of Helsinki. Subjects were recruited from among university students by advertisement. Inclusion criteria for the study were sent via e-mail to all subjects, who were then interviewed to screen out cases meeting exclusion criteria. All subjects were healthy as assessed by the interview and met the following inclusion criteria: no continuous medication, no neurologic or cardiorespiratory diseases, nonsmokers, and no pregnancy. We did not undertake screening for sleep apnea or other sleep disorders. The subjects were instructed to not consume caffeine 4 h preceding the Awake scan session and 8 h preceding the Sleep scan session. Subjects were further instructed to abstain from alcohol during the night of sleep deprivation. Sample sizes of 15 (age 26.5 ± 4.2 years, 6 females) and 12 (age 26.2 ± 4.3 years, 5 females) were used for the main analysis (Fig. 1).

### Data collection

**Figure 2.**
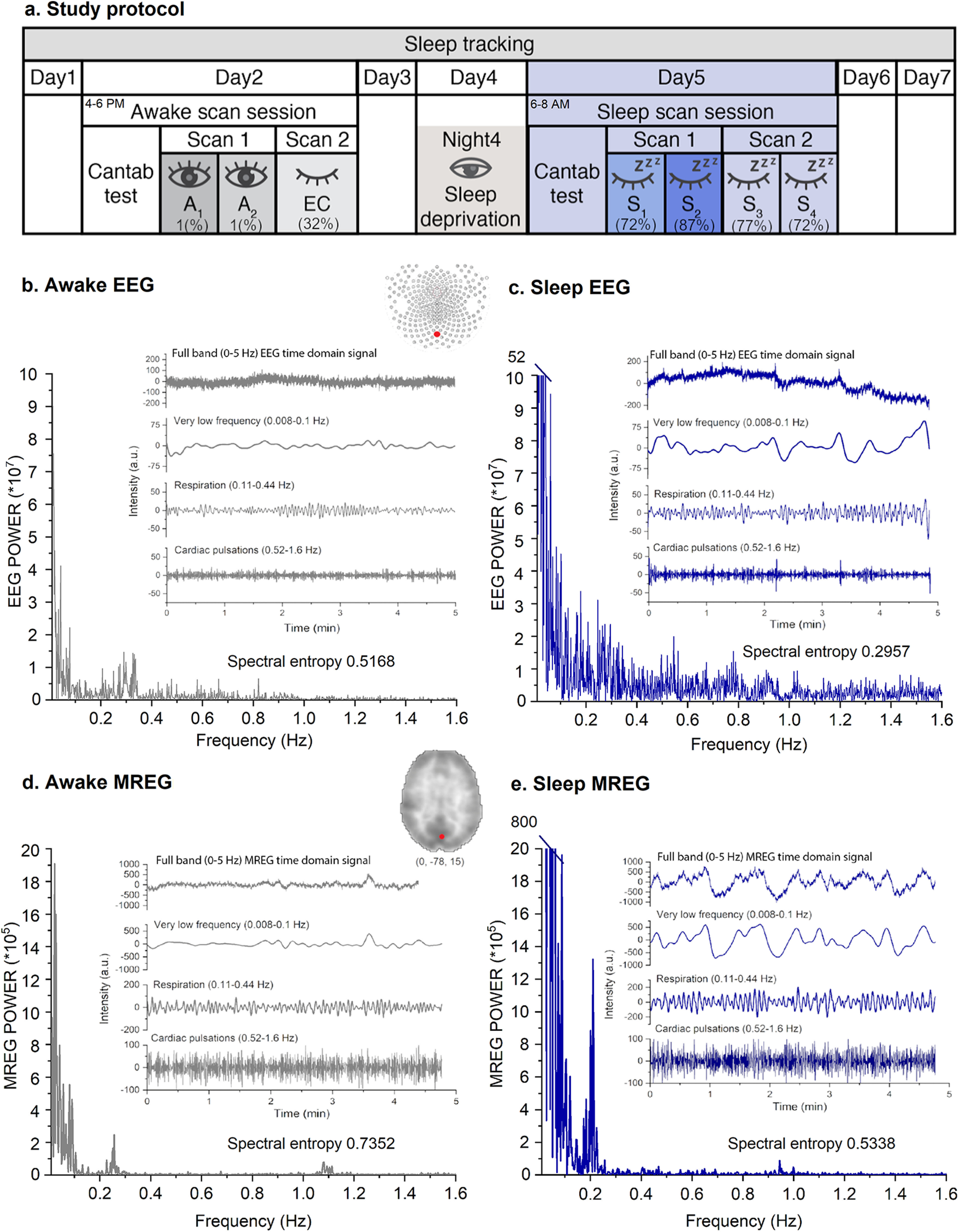
Study protocol and exemplary signals of awake state and NREM sleep. ***a***, Study design through 1 week of sleep monitoring. Awake scanning session was performed on day 2 after a good night of sleep. Sleep scanning session was performed on day 5, the morning after a night of documented sleep deprivation. At the beginning of both sessions, subjects underwent a CANTAB test to evaluate reaction times. In the Awake scan session, two scans were recorded, (1) eyes open (A_1–2_) and (2), eyes closed. In the Sleep scan session two scans were taken, (1), a 10 min Sleep scan (S_1–2_) and (2) a 10 min Sleep scan (S_3–4_). Numbers (A_1–2_, S_1–4_) correspond to 5 min segments that were treated separately for further analysis. Percentage represents the average fraction of sleep during the scan segment. ***b–e***, Examples of electrophysiological and dynamic brain pulsations measured in the same subject while awake (100% awake) and sleeping (80% NREM stage 2; 20% NREM stage 1) for 5 min recordings. Thus, the power spectra were calculated for an entire 5 min segment. The data were measured simultaneously by 256 lead high density DC-EEG Oz-channel presented in ***b***, ***c*** and fast 10 Hz magnetic resonance encephalography (MREG) (***d***, ***e***) covering the whole brain in the ≤5 Hz band. Both time and frequency domain data indicated increased power and amplitude of brain pulsations on transition from the EEG-verified fully awake state to NREM sleep. The dominant increase in very low-frequency pulsation power reshapes the power spectrum distribution and alters the spectral entropy of the EEG and MREG signals.

Subjects were scanned after a normal night of sleep (7.8 ± 1.3 h sleep the previous night) in the afternoon starting at 4:00–6:00 P.M. (and 3 d later after a monitored interval of wakefulness lasting 24 ± 1.3 h) in the early morning starting at 6–8:00 A.M. (Fig. 2*a*). We had sleep deprived the subjects, aiming to maximize the likelihood of them entering a deeper and more consolidated sleep state during the MR imaging session the next day (Kaufmann et al., 2006; Horovitz et al., 2009). At home, the sleep deprivation was verified by a smart ring (see details below). Subjects were instructed to wear the ring for at least 24 h preceding the Awake and Sleep scan sessions. The smart ring records upper limb motion (3-D accelerometer, 50 Hz), photoplethysmograms (250 Hz), and skin temperature (1/min). Validation studies for polysomnography showed 96% sensitivity for detecting sleep, 48% specificity for detecting awake state, and medical-grade actigraphy (*r* = 0.86; correlation in total sleep time), thus confirming that the ring signals adequately separate awake from sleep states (de Zambotti et al., 2017; Mehrabadi et al., 2020). Smart ring data were available for 14 of 15 scanning subjects.

All subjects were scanned in Oulu, Finland, using a MAGNETOM Skyra 3T (Siemens Healthineers) scanner with a 32-channel head coil. The subjects were scanned with the MREG fast fMRI sequence in synchrony with a previously described multimodal scanning setup (Korhonen et al., 2014). MREG is a single-shot sequence undersampling k-space with in/out stack-of-spiral trajectories in three dimensions (Zahneisen et al., 2012; Assländer et al., 2013; Lee et al., 2013). The following parameters were used for MREG: TR = 100 ms, echo time (TE) = 36 ms, FA = 5°, field of view (FOV) = 192 mm, and 3 mm cubic voxels. Parameters for three-dimensional structural T1 MPRAGE were TR = 1900 ms, TE = 2.49 ms, FA = 9°, FOV = 240 mm, and slice thickness of 0.9 mm. MREG data were reconstructed using L2-Tikhonov regularization with lambda 0.1, where the latter regularization parameter was determined by the L-curve method with a MATLAB reconstruction tool provided by the sequence developers (Hugger et al., 2011).

Earplugs were used to reduce perception of scanner noise, and head cushions restricted movement. During the Awake scan session, two MREG scans with simultaneous EEG and cardiorespiratory signals were recorded as follows: (1) 10 min resting state, Eyes open, Awake scan (A_1–2_) fixating on a cross on the screen and (2) 5 min resting state, Eyes closed (EC; Fig. 2*a*) with ambient light. Three days later during the Sleep scan session, one or two MREG sequences were recorded, (1) the first 10 min Sleep scan (S_1–2_) and (2) the second 10 min Sleep scan (S_3–4_), without ambient light, during which the subjects were allowed to fall asleep *ad libitum*. Subjects were advised to contact the staff if they were not feeling somnolent after the first scan, which resulted in termination of the session for two of 15 subjects. An anatomic MR scan was performed at the end of both sessions.

When the subject was asleep when the scanning ended, lights were turned on, and the subject was slowly woken up while remaining in the scanner. Because of individual differences in awake vigilance and ability to sleep in the MRI scanner, the 10 min scans were divided into 5 min segments (A_1_, A_2_, S_1_, S_2_, S_3_, and S_4_) to separate the awake and sleep states more effectively (Fig. 2*a*). We used 5 min segments because the accuracy of spectral analysis of very low-frequency pulsations increases as a function of the data recording length. For further analysis, we selected the segment (from S_1_ to S_4_) with the highest sleep content so that the Sleep data used for comparisons contained 91 ± 11% N1 (light sleep) or N2 (intermediate) sleep (A_1_ vs S_n_).

EEG (0.01 Hz high-pass filtering) was recorded using the Electrical Geodesics MR-compatible GES 400 system (Magstim), with a 256-channel high density net. Electrode impedances were <50 kΩ and the sampling rate was 1 kHz (Data from six subjects were sampled with 250 Hz by mistake, from which two—one A_1–2_ and one EC—EEG datasets were not usable for sleep scoring). Before scanning, signal quality was tested outside the scanner room by recording 30 s epochs of EEG with eyes open and eyes closed and by visually checking all the channels. Respiratory belt and fingertip peripheral SpO_2_ and anesthesia monitor data [ECG, fingertip peripheral SpO_2_, and end-tidal carbon dioxide (EtCO_2_); Datex-Ohmeda S/5 Collect software] were measured in synchrony with the EEG, as described previously (Korhonen et al., 2014).

Before the imaging, the subjects performed the reaction time and paired-associate learning tests from Cambridge Neuropsychological Test Automated Battery (CANTAB; https://www.cambridgecognition.com/) on a tablet computer. The CANTAB test is a widely accepted standard for measuring cognitive state and vigilance level.

### Preprocessing and analysis of MREG data

After reconstruction, MREG data were preprocessed and analyzed using Functional MRI of the Brain Software Library (FSL; Brain Extraction Tool, version 5.09; Jenkinson et al., 2012; Smith, 2002), Analysis of Functional NeuroImages (AFNI, version 2; Cox, 1996) and MATLAB (R2019). The brain was extracted from structural 3D MPRAGE volumes using neck cleanup and bias field correction options (Smith, 2002). The functional data preprocessing was performed using the FSL pipeline. Data were high-pass filtered with a cutoff frequency of 0.008 Hz, and framewise head motion was then performed with FSL 5.08 MCFLIRT (Functional MRI of the Brain Linear Image Registration Tool; Jenkinson et al., 2002). MCFLIRT relative mean displacement values (mm) of scan segments (A_1_ vs EC, S_1_, S_2_, S_3_, and S_4_) and Awake versus Sleep analysis (A_1_ vs S_n_) did not differ significantly (paired *t* test, p_all_ > 0.11). Spatial smoothing was performed with fslmaths using a 5 mm Full Width at Half Maximum (FWHM) Gaussian kernel. The highest spikes in the MREG data time series were removed using the 3dDespike function in AFNI. Data were then registered with the Montreal Neurologic Institute (MNI)152 space at 3 mm resolution for comparable analysis among subjects. One hundred time points (10 s each) were excluded from the beginning of both 5 and 10 min data segments to reach steady-state signal saturation. Accordingly, from the 10 min data, the final 100 time points were extracted to get a time series of the same length. Finally, we generated datasets for 2861 time points (A_1_–S_4_), which corresponds to ∼5 min.

### Preprocessing and analysis of EEG data

We used standard EEG cleanup strategies and EEG sleep state scoring for datasets of the 15 subjects in whom we had simultaneously measured EEG and MREG. Because of instances of noisy EEG signals that were unsuitable for sleep scoring in three subjects, the final sample size in most of the MREG analysis was 12 (paired *t* test, A_1_ vs S_n_). In EEG slow oscillation analysis, sample size was 10 versus 12, because 2 of 12 had no usable awake EEG. Data of all 15 subjects were used for calculating the accuracy of spectral entropy sleep detection (see below, MREG signal spectral entropy analysis).

EEG recordings were preprocessed using the Brain Vision Analyzer (version 2.1, Brain Products) after file format conversion via Brain Electrical Source Analysis Research (version 7.0). Gradient artifacts because of static and dynamic magnetic fields present during the MRI data acquisition and ballistocardiographic (BCG) artifacts were corrected using the average artifact subtraction method (Allen et al., 1998, 2000). The absence of gradient of BCG artifacts was verified by visual inspection. After preprocessing, EEG data were visualized according to the 10–20 system instructions for sleep state scoring. Two experienced specialists in clinical neurophysiology, who were trained in EEG sleep scoring, performed the scoring in 30 s epochs according to American Academy of Sleep Medicine (AASM) 2017 guidelines for clinical sleep studies, and the final decisions on sleep states were obtained by consensus. EEG epochs were scored as awake, N1 (light sleep), N2 (intermediate sleep with sleep spindles and/or K-complexes), N3 [slow-wave activity (SWA)], or REM (sleep with rapid eye movements).

For slow oscillation power spectral analysis, we removed linear trends to exclude stable conductivity changes at the electrode–skin interface, which could otherwise have affected the performance of various processing steps. We used fast independent component analysis (FastICA) in combination with principal component analysis dimension reduction (150 components) to transform the datasets into independent components, where artificial ICs were identified and removed with the help of the EEGLAB SASICA (SemiAutomatic Selection of Independent Components for Artifact correction in the EEG) tool after visual confirmation. To furthermore minimize nonlinearities and enhance the performance of ICA, we used a spike detection algorithm to remove the most prominent artifactual signal excursions. To keep the recordings intact, we interpolated the trends for the gaps and used data from intact signal epochs to fill the gap in the manner similar to inpainting. Bad channels removed before ICA were afterward reintroduced using spherical interpolation. Recordings were referenced to linked mastoid electrodes, which were located near other electrodes but record less brain activity. Next, we generated 5 min segments (A_1_–S_4_) corresponding with the analyzed MREG data. Power estimates were then calculated using short-time Fourier transform combined with a Hamming window of 30 s length and 50% overlap. We calculated relative power (RP) to attenuate the subject specific variability RP_sw_ = P_sw_/P_tot_. The time average was used to gain one RP estimate for each subject. As previous studies have linked increased CSF flow to slow-wave changes (Fultz et al., 2019), we chose to study the SWA (0.5–4 Hz) and slow oscillations (0.2–2 Hz). Our direct current EEG allowed us to evaluate frequencies <0.5 Hz. To follow the previous literature, we set 0.2 Hz as the lower limit for slow oscillations as these are known to increase in amplitude during N2 sleep (Brancaccio et al., 2020). The upper limit was chosen to meet the standard requirement in scoring of slow-wave sleep.

### MREG signal spectral entropy analysis

Spectral entropy from EEG and EMG is known to measure depth of anesthesia (Viertiö-Oja et al., 2004) and sleep (Mahon et al., 2008) and to correlate with the level of consciousness. Given the 10 Hz sampling rate of MREG data, we supposed that calculation of its spectral entropy might present an alternative to EEG spectral entropy analysis by showing similar reductions during sleep (Vakkuri et al., 2005; Mahon et al., 2008; Kumar et al., 2013). We calculated a single spectral entropy value for whole time series with MATLAB (R2019) using the pentropy function, which produced a spatial map. The method is based on treating the normalized Fast Fourier Transform (FFT) power spectrum as a probability distribution, which is then used to calculate the Shannon entropy (Shannon, 1948). The spectral entropy value describes the irregularity or complexity of the signal, where a noiseless, regular, and totally predictable signal such as a perfect sine wave has an entropy value of zero, and complex signals with several prominent frequency bands have a higher spectral entropy value. Thus, spectral entropy tends to decrease when the power in one frequency range starts to dominate.

We tested the accuracy of spectral entropy to discriminate sleep from wakefulness. First, we created a model that included data from only the awake and deepest sleep conditions. As there were 30 fully awake 5 min segments, we chose for comparison the 30 segments with the deepest sleep state (88 ± 12% N1 or N2). We weighted the sleep states incrementally with increasing depth of sleep (awake = 0, N1 = 1, N2 = 2, N3 = 3), multiplied the weighting values by the corresponding number of 30 s epochs, and calculated the sum to get an EEG-weighted sleep score for each 5 min segment. Thus, a fully awake 5 min session corresponds to a minimum EEG weighted sleep score of zero and a 5 min segment of entirely N3 sleep corresponds to a maximum score of 30. Data were included when sleep states and spectral entropy were both available (A_1_, *n* = 12; A_2_, *n* = 12; EC, *n* = 13; S_1_, *n* = 12; S_2_, *n* = 12; S_3_, *n* = 10; and S_4_, *n* = 10).

**Figure 3.**
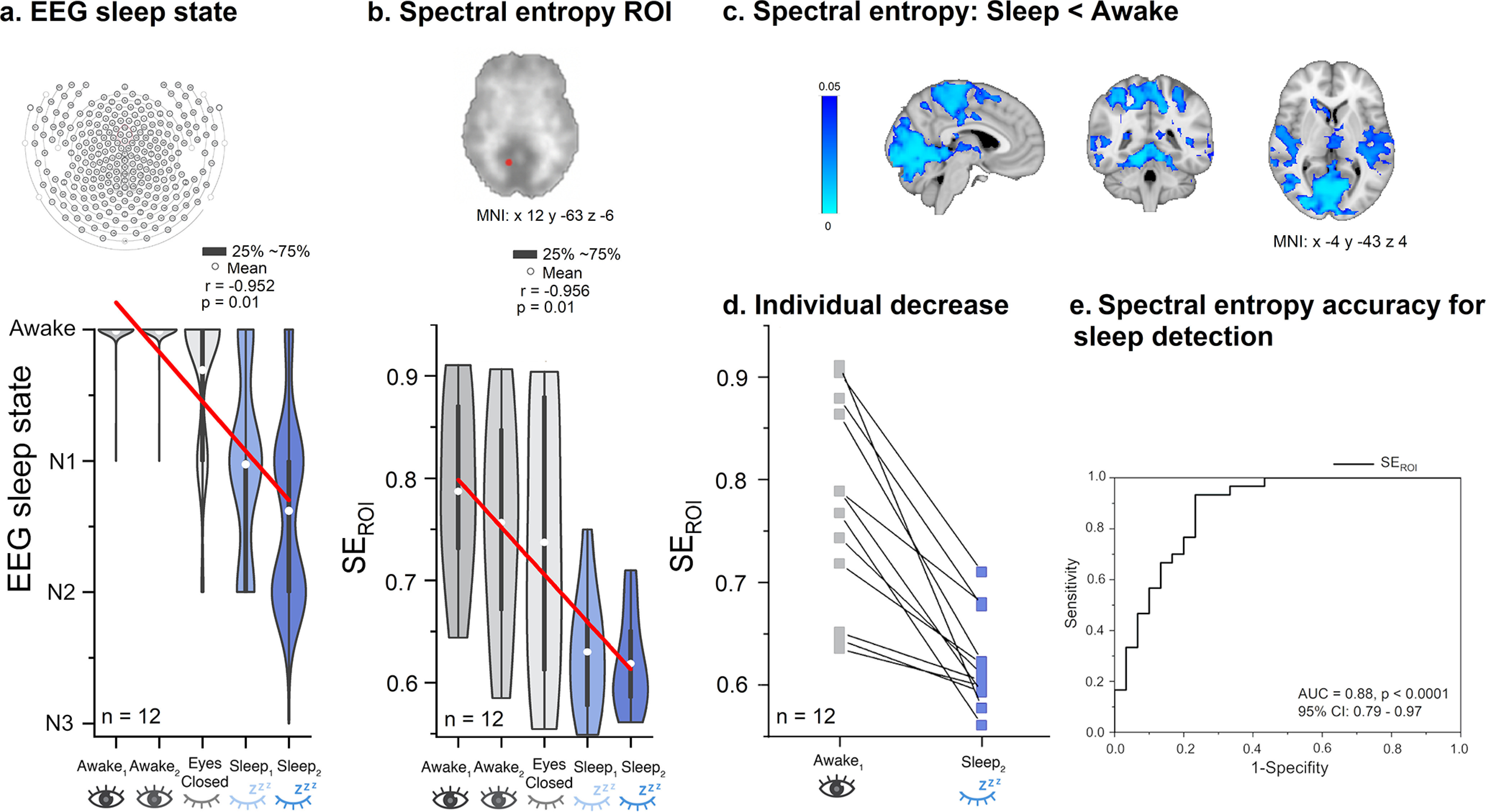
Spectral entropy of MREG decreases in line with increased amount of NREM sleep. ***a***, EEG sleep state data show that the amount and depth of sleep both increase as a function of scan segment. ***b***, Visual cortex SE_ROI_ spectral entropy of region of interest (visual cortex, SE_ROI_) of MREG data predicts sleep and wakefulness across subjects (*n* = 12). Results indicate linear declines both in EEG sleep state and MREG SE_ROI_ values as a function of scan segment in the experiment. ***c***, Spectral entropy decreased significantly in posterior brain areas (*p* < 0.05, df 11). ***d***, The SE_ROI_ showed a drop in all subjects in the transition from wakefulness to EEG-verified sleep states in S_2_. ***e***, The receiver operating curve (ROC) curve of SE_ROI_ data indicate high accuracy, area under the curve (AUC) = 0.88 (*p* < 0.0001), in the ability to distinguish sleep (*n* = 30 Sleep segments) from awake data (*n* = 30 Awake segments). The model has a sensitivity of 93% and a specificity of 77%. *p*-value, p; Correlation coefficient, r; Montreal Neurologic Institute, MNI; Confidence interval, Cl.

To test the fitness of spectral entropy to accurately identify sleep, we chose a region of interest (ROI) from visual cortex (spherical ROI of 5 mm radius, MNI × 12 year −63 *z* −6) for further analysis, as the fMRI signal in this region robustly changes across the sleep–wake cycle (Fukunaga et al., 2006; Chang et al., 2016; Liu et al., 2018). We then compared AASM sleep scores and the ROI spectral entropy results to see whether spectral entropy changes occurred before sleep onset (Fig. 3*a*,*b*). Next, we calculated the receiver operating curve (ROC) to determine the separability, sensitivity and specificity of the ROI spectral entropy to distinguish sleep from wakefulness.

We then performed a voxel-level analysis to map regions in which spectral entropy distinguished between Awake versus Sleep states (A_1_ vs S_n_; *n* = 12 for whom sleep state was available). Next, we applied a bandpass filter with 3dTproject for very low (0.008–0.1 Hz), respiratory (0.11–0.44 Hz), and cardiac (0.52–1.6 Hz) frequencies to enable a comparison of spectral entropy in relation to these distinct physiological phenomena. The very low range of 0.008–0.1 Hz was chosen to obtain maximum possible coverage of the low frequencies, without cross-talk from respiratory frequencies. Group mean ranges for respiratory and cardiac frequencies were verified from individual anesthesia and/or scanner physiological monitoring signals for FFT analysis. As previously shown, cardiorespiratory distributions in MREG correspond to the frequency ranges in physiological signals (Tuovinen et al., 2020). First, we defined the peak of the respiratory frequency and then identified the starting and ending points of the respiratory power (lowest point around the peak) from the EtCO_2_ signals; if not available, we used data from the respiratory belt of each scan separately. All subjects had some data available (A_1_–S_4_) but in 7 of 60 of the datasets we could not make the definition. Cardiac frequency range was defined similarly from the fingertip peripheral SpO_2_ (8/60 missing, but at least one dataset was available for all subjects). Group mean ranges were chosen based on the lowest and highest values of the individual ranges, with no overlap of other frequency bands.

### Power spectral analysis of MREG signal

Using power spectrum analysis, we studied differences between A_1_ versus S_n_, voxel-wise and for the global signal, which is known to shift depending on state of arousal (Chang et al., 2016; Wen and Liu, 2016; Liu et al., 2018). Power spectral analysis is an effective tool to separate different sources of brain fluctuations according to different frequency ranges (Duff et al., 2008). Here, we separated three frequency bands as described in the preceding section. For each frequency range, we calculated an FFT power density map with the 3dPeriodogram function in AFNI for the A_1_ and S_1–4_ scans. The scanning segments together comprised 2861 time points, and FFT was conducted with 4096 bins so that we ended up with 2048 bins corresponding to 0–5 Hz (0.0024 Hz resolution). Very low-frequency, respiratory, and cardiac verified FFT power frequency ranges were separated from the global and voxel-wise MREG periodograms using fslroi (after specifying the frequency ranges), and the summed power over each frequency range was calculated using 3dTstat.

### Correlation analysis between spectral entropy and power

Using fslcc, spectral entropy difference maps were correlated with the corresponding sum of power maps to evaluate the extent of their spatial overlap. The three frequency band results of both methods were likewise correlated to reveal whether the different pulsations involved similar brain regions.

### Statistical analysis

We used FSL randomize with threshold-free cluster enhancement and familywise error (false positive) control along with a two-sample paired *t* test model (A_1_ vs S_n_, df 11) in the voxel-wise spectral entropy and the FFT power analyses. We calculated Pearson correlations for sleep state change and spectral entropy ROI in relation to scan segments (from A_1_ to S_2_).

The ROC curve was calculated to reveal the accuracy of spectral entropy to distinguish sleep and awake states, and the optimal sensitivity and specificity of the model was calculated (MATLAB R2019). For smart ring activity, CANTAB data, and head motion data, statistical differences were calculated by a two-sample paired *t* test model using IBM SPSS Statistics 26,0. In the EEG slow oscillation analysis, statistical testing for the difference in means was made with permutation testing by randomly shuffling the group labels 10,000 times. We transformed the differences into *z* scores, from which statistically significant (two tailed, *p* < 0.05) results can be obtained.

## Results

We studied spectral entropy and power sum of physiological brain pulsations during awake and NREM sleep and then investigated their relation to EEG slow oscillation power. We performed main analysis with a sample size of 12 or 15 (Fig. 1). Data from a representative subject demonstrates the dramatic effect of NREM sleep on the power intensity of the EEG (Fig. 2*b*,*c*) and MREG data in the three extracted pulsation frequency ranges (Fig. 2*d*,*e*). Respiratory and very low-frequency power increased sharply, and spectral entropy decreased in NREM sleep (the chosen 5 min segment here includes 80% N2 and 20% N1 sleep), especially when entropy was calculated in the visual cortex (Fig. 2*b–e*).

### N1 and N2 states occurred as majority in sleep scans

Sleep scoring was performed for 15 subjects across the Awake and Sleep scan sessions. Only a single epoch of sleep was detected for one individual in the Awake eyes open (A_1–2_) scan sessions. Among the Eyes closed scans, 32% were scored as NREM sleep, most of which (∼88%) were N1. Finally, the Sleep scan sessions (S_1–4_) included 72–87% N1 or N2 sleep (Table 1, Fig. 2*a*).

**Table 1.** The amount of NREM sleep was higher during the sleep scan session than in the awake scan session

	Awake scan session			Sleep scan session			
Mean ± STD	A_1_	A_2_	EC	S_1_	S_2_	S_3_	S_4_
(%)	*n* = 12	*n* = 12	*n* = 13	*n* = 12	*n* = 12	*n* = 10	*n* = 10
Wake	99 ± 3	99 ± 3	68 ± 39	22 ± 19	11 ± 23	23 ± 32	22 ± 35
N1	1 ± 3	1 ± 3	28 ± 39	47 ± 25	40 ± 33	40 ± 22	35 ± 30
N2	0	0	4 ± 14	25 ± 19	46 ± 38	37 ± 29	37 ± 36
N3	0	0	0	0	1 ± 3	0	0
No REM sleep was observed.							
NREM sleep (%)	1 ± 3	1 ± 3	32 ± 40	72 ± 17	87 ± 21	77 ± 30	72 ± 35

Mean ± SD (%) of the amounts of wakefulness, NREM, and total sleep times were calculated from the number of sleep-scored EEG epochs in each condition. The highest amount of sleep was achieved in S2 (mean across subjects 87%). Unknown epochs are likely because of artifacts. A_1_, Awake 1 time segment, 5 min; A_2_, awake 2 time segment, 5 min; EC, 5 min; S1, sleep 1 time segment, 5 min; S2, sleep 2 time segment, 5 min; S3, sleep 3 time segment, 5 min; S4, sleep 4 time segment, 5 min. The *n* values represent the number of a total of 15 subjects with an available EEG for sleep scoring.

**Table 2.** Respiratory and cardiac frequency ranges were determined from physiological EtCO_2_ and SpO_2_ signals

		Minimum	Peak	Maximum
Respiratory EtCO_2_ (Hz)				
Eyes open	*n* = 14	0.17 ± 0.06	0.27 ± 0.06	0.35 ± 0.05
Eyes closed	*n* = 13	0.13 ± 0.06	0.24 ± 0.06	0.32 ± 0.05
Sleep scan 1	*n* = 14	0.17 ± 0.05	0.24 ± 0.04*	0.31 ± 0.05
Sleep scan 2	*n* = 12	0.16 ± 0.05	0.24 ± 0.05*	0.32 ± 0.06
Cardiac SpO_2_ (Hz)				
Eyes open	*n* = 14	0.96 ± 0.10	1.07 ± 0.12	1.19 ± 0.16
Eyes closed	*n* = 13	0.90 ± 0.12	1.03 ± 0.13***	1.19 ± 0.18
Sleep scan 1	*n* = 14	0.81 ± 0.13	0.94 ± 0.16**	1.09 ± 0.17
Sleep scan 2	*n* = 11	0.82 ± 0.12	0.94 ± 0.16	1.11 ± 0.21

Individually determined minimum and maximum values for lower and upper edge of the power peak were used for further analysis. Respiratory rate and heart rate decreased in NREM sleep versus awake based on the power peaking value. The *n* values represent the number of a total of 15 subjects with an available end-tidal carbon dioxide (EtCO2) and fingertip peripheral (SpO2) signals.

*p < 0.05;

**p < 0.01;

***p < 0.001.

To investigate effects of NREM sleep on MREG-detected brain pulsations, we selected individual 5 min segments that included the highest amount of sleep. For subject level analysis (*n* = 12), the average amount of NREM sleep in the Sleep scan session was 91 ± 11% (N1, 33 ± 28%; N2, 57 ± 32%; N3, 1 ± 3%; awake, 8 ± 1%; unknown, 1 ± 3%), whereas the awake scan (A_1_) consisted of wakefulness and only 1 ± 3% N1 sleep (Table 1).

### Frequency ranges for pulsation analysis were determined from physiological EtCO_2_ and SpO_2_ signals

Group mean ranges for respiratory and cardiac frequencies were obtained from individual physiological monitoring EtCO_2_ or respiratory belt and SpO_2_ signal data based on the minimum and maximum points of the highest peak (Table 2). The peaking value was significantly lower for EtCO_2_ in S_1_ (*p* = 0.012) and S_2_ (*p* = 0.029) when compared with eyes open (EO) and for SpO_2_ in EC (*p* = 0.0009) and S_2_ (*p* = 0.001), all when compared with EO.

### Smart ring corroborated sleep deprivation, and CANTAB revealed slower reaction time after sleep deprivation

Based on pilot data for the successful measurement of synchronous multimodal MREG and DC-EEG signal (Korhonen et al., 2014), we recruited healthy subjects for a sleep monitoring study and requested them to use the ring for at least 24 h preceding the Awake scan session and likewise to verify sleep deprivation preceding the Sleep scan session. Based on the smart ring data, we observed only 9 ± 19 min of sleep before Sleep scan sessions, corroborating that our subjects were indeed sleep deprived at that time (14 of 15 subjects). Three of 15 subjects had incomplete smart ring data during the night of sleep deprivation (16, 25, and 57 min) and nine of 15 subjects had some missing data during the entire 24 h before the scan. After sleep deprivation, the subjects exhibited significantly increased reaction times, indicative of reduced vigilance compared with their results during the Awake scan session following a normal night of sleep (CANTAB reaction time test, 429 ± 29 > 406 ± 30 ms, *p* = 0.028).

### Full band spectral entropy of MREG decreased during NREM sleep

We focused our analysis of MREG spectral entropy in an ROI placed in the visual cortex (Fig. 3), a brain area known to show changes in very low-frequency pulsations during periods of decreased vigilance and sleep (Fukunaga et al., 2006; Chang et al., 2016; Liu et al., 2018). Thus, we evaluated whether MREG spectral entropy results in visual cortex can distinguish Awake and Sleep segments. As expected, the weighted sleep staging data (awake, N1, N2, N3) showed linear relationships with the scan segments (A_1_–S_2_; *r* = −0.952, *p* < 0.01), closely resembling the association observed between the MREG spectral entropy ROI data and the scan segments (*r* = −0.956, *p* < 0.01; Fig. 3*a*,*b*,*d*). The accuracy whereby spectral entropy in the visual cortex ROI separated sleep from wakefulness was further investigated using ROC analysis. By comparing Awake segments (*n* = 30, fully awake) against Sleep (*n* = 30, 88 ± 12% NREM sleep), we were able to separate sleep from wakefulness with high accuracy [Area Under the Curve (AUC) = 0.88, *p* < 0.0001; Fig. 3*f*], with a sensitivity of 93% and specificity of 77%. These results show that spectral entropy, which is a marker of vigilance, indeed reduced with increasing NREM sleep depth.

To analyze spatial differences in entropy between Awake and Sleep, we calculated full band voxel-wise maps in the Eyes open (A_1–2_) and Sleep segments (S_1_–_4_). We chose the A_1_ segment for the analysis because of the highest awake state (99% awake, and highest vigilance based on spectral entropy ROI). We compared the Awake data versus the time series with the highest proportion of NREM sleep (91 ± 12% NREM sleep, A_1_ vs S_n_, df 11). The occipital, parietal, and temporal lobes and cerebellum exhibited significant reductions in spectral entropy (*p* < 0.05, df 11; Fig. 3*c*) during NREM sleep, whereas parts of the frontal lobe did not show spectral entropy changes.

### Global MREG signal revealed enhanced whole-brain pulsatility

**Figure 4.**
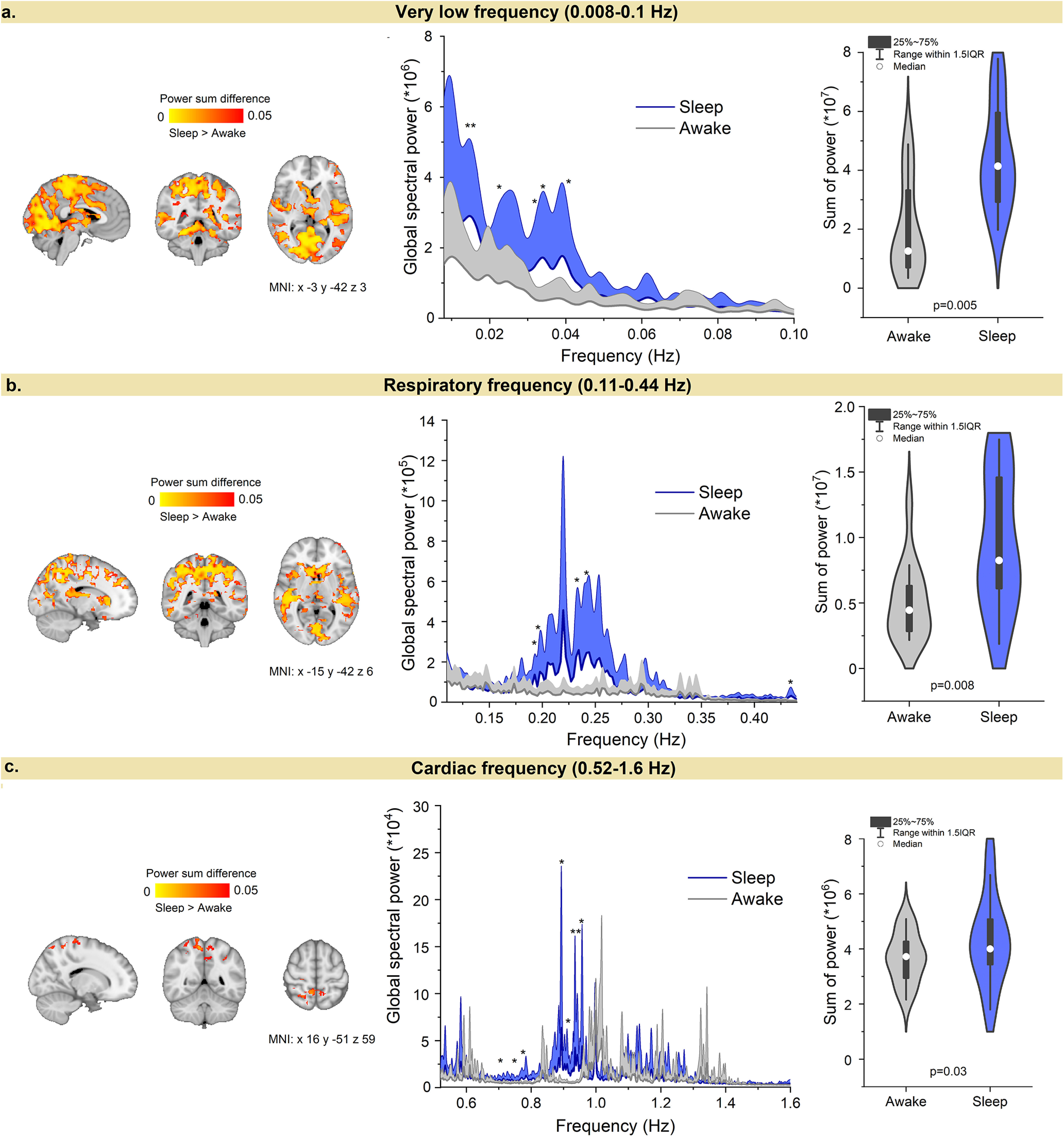
NREM sleep sharply changes the power spectrum of physiological brain pulsations. ***a–c***, Global MREG power spectra and power sum difference (paired *t* test) between Awake and Sleep in ***a*** very low frequency (0.008-0.1 Hz); ***b***, respiratory frequency (0.11–0.44 Hz); and ***c***, cardiac frequency (0.52–1.6 Hz) bands. The global very low and respiratory frequency power both increased markedly in sleep, and both differed significantly in posterior brain regions. The global cardiac frequency power increased to a lesser extent but showed a decrease in heart rate (*p < 0.05, df 11; **p < 0.01). Montreal Neurologic Institute, MNI.

Next, we investigated whether the reduction in spectral entropy during NREM sleep might be linked to an increase in brain pulsation strength. FFT power of the whole-brain MREG signal was calculated to assess the strength of each pulsation. As global fMRI is known to reflect arousal fluctuations (Chang et al., 2016; Wen and Liu, 2016; Liu et al., 2018), we first calculated the summed power for the global MREG spectrum. As hypothesized, the spectral power increased markedly in the very low and respiratory frequencies, and to a lesser extent in the cardiac pulsation range (Fig. 4). Together, the power in each of the three physiological frequency bands revealed significant increases during NREM sleep compared with waking, suggesting that physiological brain pulsations are globally enhanced during sleep.

### Sleep-induced entropy and power changes occurred mainly in very low and respiratory pulsations

**Figure 5.**
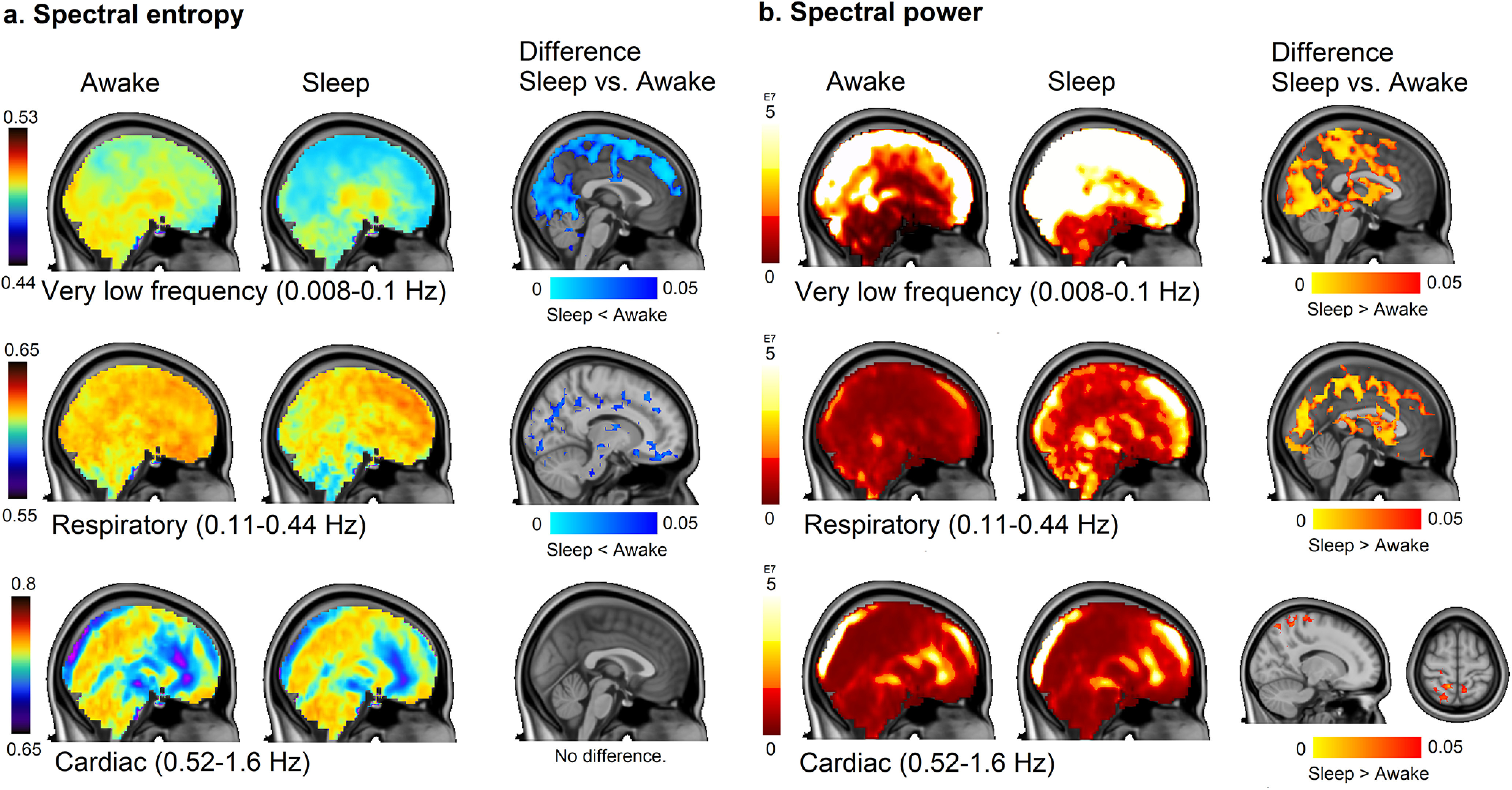
Spatial distribution of spectral brain pulsation entropy and power are linked to frequency band. Paired *t* test shows differences in Sleep versus Awake state (*p* < 0.05, df 11). Thresholded mean maps across subjects and difference maps are presented. ***a***, ***b***, Spectral entropy (***a***), spectral power (***b***) in each pulsation frequency band in 5 min segments in NREM sleep. The three rows show results in very low, respiratory, and cardiac frequencies, respectively. The spatial overlap of the power and entropy increases dimishes as a function of frequency, with the largest overlap in the very low-frequency range, partial overlap in the respiratory band, and no overlap in cardiac frequencies (no change in spectral entropy).

To investigate the spatial distribution of brain pulsations, we compared spectral power and spectral entropy maps between Awake and Sleep states, which showed significant differences mainly in very low and respiratory frequency ranges. As the power increased in very low, respiratory, and cardiac frequency bands in the transition to sleep, spectral entropy decreased in very low and respiratory bands. Figure 5 shows spectral entropy and power changes across all brain voxels for evaluating the relative contributions of the three physiological brain pulsations during Awake and Sleep conditions.

The largest state-dependent change in power and spectral entropy occurred in the very low-frequency band extending over posterior parts of the brain. The correlation coefficient between power and spectral entropy differences in Awake versus Sleep (*r* = 0.45) indicated widespread overlapping regions, especially in posterior parts of the brain. There was a power increase with no decrease in entropy in the brainstem and ventricles, and unchanged power along with low entropy values in frontal cortex. Thus, entropy can change without concomitant power changes.

The respiratory pulsation power increased, and its spectral entropy decreased significantly during sleep (*p* < 0.05, df 11; Figs. 4, 5). The power increase was located in upper posterior brain areas and in the ventricles, overlapping with the regions showing a very low-frequency increase (*r* = 0.34; Figs. 4, 5). The most significant power increase occurred in typical Awake activation areas (Downar et al., 2000), thus involving the primary sensorimotor and auditory cortices, along with the V1–V2 visual areas. Power also increased in the CSF filled spaces of the lateral and central sulci, indicating strong respiratory pulsatility in sleep. In contrast to the increased respiratory frequency power, the decreased respiratory frequency spectral entropy was located more toward the basal brain structures and in the frontal pole, with less overlap with the power changes seen in upper posterior regions, although still showing some overlap in visual and auditory cortices (*r* = 0.2). Spectral entropy changes were found subcortically near the gray matter/white matter border and more prominently in white matter, which has greater venous than arterial circulation. There was decreased spectral entropy in the brainstem, which did not overlap with power changes. Overall, very low and respiratory frequency power bands showed widespread increase during NREM sleep, in conjunction with decreased entropy in partially overlapping brain areas.

The power of cardiac pulsation increased in somatosensory cortex during sleep (*p* < 0.05, df 11; Fig. 5*b*, bottom), but spectral entropy did not change in the cardiac frequency range. Overall, we found that power increased in widespread brain regions for all three frequency ranges, whereas spectral entropy decreased in very low and respiratory frequency bands during NREM sleep.

### EEG slow oscillation changes overlapped with very low, respiratory, and cardiac pulsation changes of MREG

**Figure 6.**
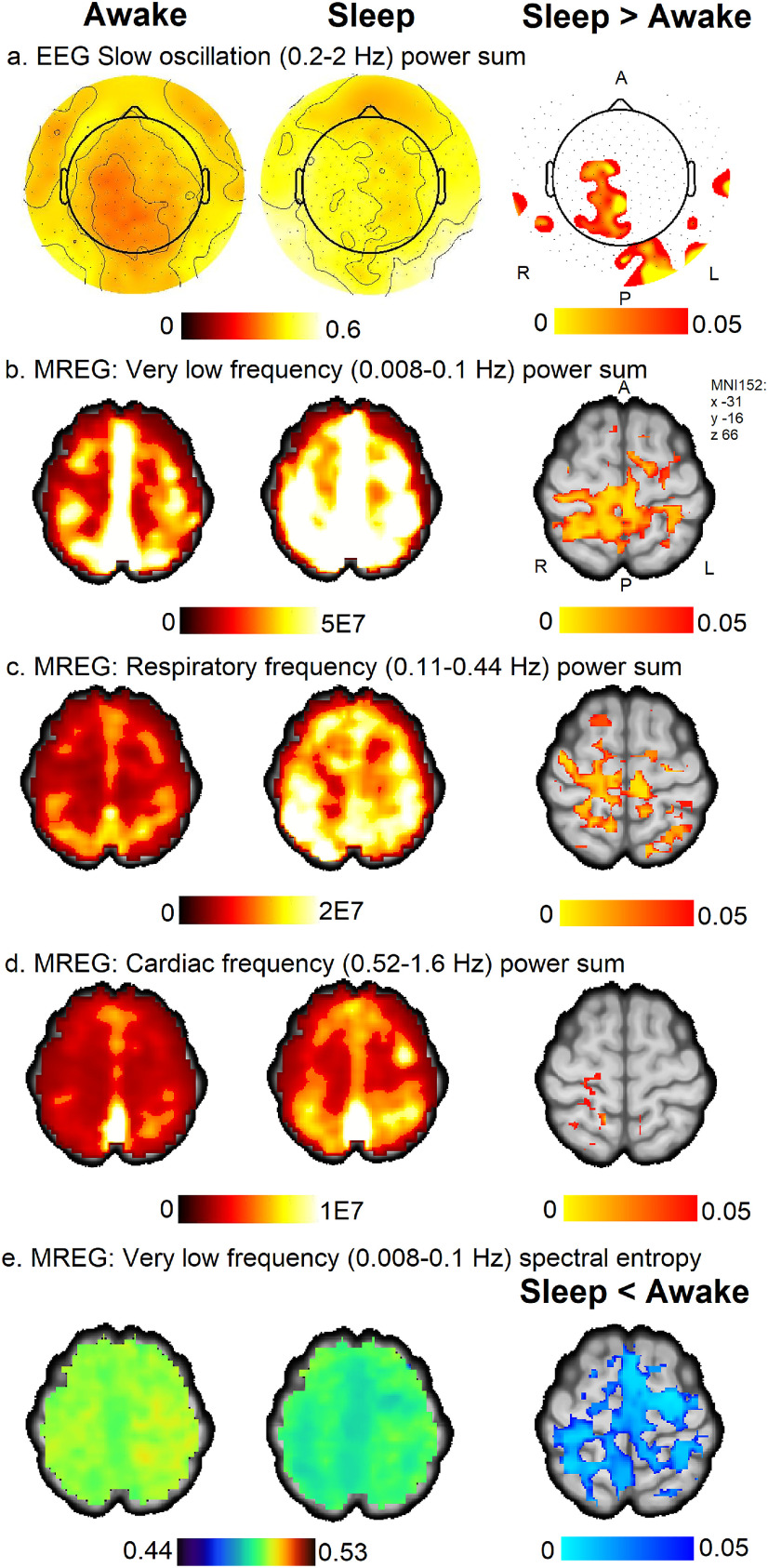
EEG slow oscillation (0.2–2 Hz) power increased in spatially overlapping regions in very low, respiratory, and cardiac frequency pulsations in MREG. ***a***, EEG slow oscillation (0.2–2 Hz) power sum mean maps in Awake and Sleep and difference (Sleep > Awake, *p* < 0.05). EEG power maps were converted to the radiologic perspective for comparison with MREG results. ***b–d***, Power sum of MREG pulsations in very low frequency (0.008–0.1 Hz; ***b***), respiratory frequency (0.11–0.44 Hz; ***c***), and cardiac frequency (0.52–1.6 Hz; ***d***); Sleep > Awake, *p* < 0.05. ***e***, Spectral entropy of MREG in very low frequency (0.008–0.1 Hz), Sleep < Awake, *p* < 0.05. Mean maps among subjects during Awake and Sleep are on the left, and Difference maps are in the right column.

Next, we studied whether pulsation power increase and spectral entropy decrease are associated with the SWA (0.5–4 Hz) power and slow oscillation (0.2–2 Hz) power of EEG, as delta power changes have previously been connected with BOLD signal and CSF flow (Fultz et al., 2019) and with increased β-amyloid clearance (Hablitz et al., 2019). The sleep-induced EEG-derived slow oscillation sleep changes were located in the right midline parietal and occipital areas (Fig. 6). These changes spatially overlapped with regions of increased power of all physiological brain pulsations having reduced spectral entropy. The highest overall overlap was seen in EEG slow oscillation frequency and the MREG respiratory frequency range. We did not find significant changes in SWA (0.5–4 Hz) power.

## Discussion

We studied spectral entropy and power sum changes in MREG data in very low, respiratory, and cardiac frequency bands during NREM sleep and while awake, and examined spatial similarities with concomitant EEG slow oscillation power changes. To our knowledge, this is the first study to report brain-wide enhancement and reduced spectral entropy in very low and respiratory frequency pulsations, and to a lesser extent, increased cardiac frequency power. We demonstrate that regional neurophysiological changes in EEG slow oscillation power specifically colocalize with the same regions as MREG changes for all three physiological pulsations. The interaction between 0.05 Hz CSF pulsations with neural and hemodynamic oscillations has been shown to increase during human sleep (Fultz et al., 2019), which is supported by our present observation of these changes in mainly N2 sleep. Together with previous literature, our new observations suggest that the hydrodynamic properties governing CSF movement in the brain are regulated by physiological pulsations across the sleep–wake cycle.

### Vasomotor pulsations dominate during NREM sleep

Present results are in accord with earlier literature on the conventional BOLD signal, which showed increased very low-frequency fluctuations in posterior brain regions during light sleep and episodes of low waking vigilance (Fukunaga et al., 2006; Horovitz et al., 2008; Chang et al., 2016; Liu et al., 2018). Moreover, slow deactivation cycles of the cholinergic neurons of the nucleus basalis of Meynert have been shown to precede widely distributed hemodynamic BOLD signal increase in posterior brain regions (Liu et al., 2018). Present results show increases in very low-frequency signals in posterior brain regions virtually identical to those reported by Liu et al. (2018), Spectral entropy of the EEG signal has been shown to decrease with sleep depth, where the frequency range moves toward lower frequencies and obtains a nadir during deepest sleep (Mahon et al., 2008). We found a similar pattern in MREG recordings, notably in the cerebral cortex. Therefore, we propose that the mechanism underlying the enhanced and stabilized very low-frequency signal must start to dominate in the MREG 0–5 Hz frequency range.

Low frequency BOLD fluctuations are widely attributed to functionally connected hemodynamic oscillations that are tightly coupled to neuronal activity during wakefulness (Hutchison et al., 2013; Korhonen et al., 2014). Part of this low-frequency activity at ≤0.1 Hz has been ascribed a vascular origin arising from vasomotor waves or slow sinusoidal hemodynamic oscillations (Biswal et al., 1995; Kiviniemi et al., 2000; Wang et al., 2008; Rayshubskiy et al., 2014; Kiviniemi et al., 2016). As similarly shown during N2 sleep by Özbay et al. (2019), peripheral vasomotor constrictions driven by central K-complexes precede the global BOLD signal by some 11 s in human brain. Vasomotor waves are slow, seemingly spontaneous undulations in the arteriolar wall tension that control vessel wall pulsatility and local flow resistance, which consequently influence perfusion in downstream vascular territories (Preiss and Polosa, 1974). The force of the driving pulsation depends on various factors, including the circulatory perfusion pressure and elasticity of the vessel wall, both of which are regulated by smooth muscle vasomotor tonus in the arterial wall (Hadaczek et al., 2006). Recent data have indicated that vasomotor waves can move injected tracers along the perivascular space, thus serving as a glymphatic driver (van Veluw et al., 2020).

Hillman and colleagues have detected two distinct very low-frequency phenomena using wide-field optical imaging in mice (Ma et al., 2016). Urethane anesthesia produces similar delta power activity as ketamine/xylazine anesthesia (Chauvette et al., 2011), which has been linked to increased glymphatic influx (Hablitz et al., 2019) of a similar magnitude as that occurring during natural sleep (Xie et al., 2013). A 0.04 Hz fluctuation present only in anesthesia data overshadowed the faster hemodynamic changes coupled to spontaneous neuronal activity (Ma et al., 2016), and only on removing the 0.04 Hz vasomotor fluctuation did the underlying neuronal activity-coupled and functionally connected hemodynamic signal emerge. Interestingly, we also found a peak power value at 0.04 Hz in the global MREG (Fig. 4), which might arise from a similar source. Emergence of this new peaking frequency range during sleep likely decreased the spectral entropy. Plausibly, the very low-frequency BOLD signal might originate from non-neuronal sources such as vasomotor activity, especially during sleep.

### Respiratory power increases and spectral entropy decreases during NREM sleep

For the first time, we present evidence that sleep-specific brain pulsations change in rhythm with the respiratory frequency, with increased power and reduced complexity. Although physiological pulsations can affect the BOLD signal, this interaction has not been widely studied because of technical temporal limitations of conventional fMRI (Kiviniemi et al., 2016; Huotari et al., 2019). The present acquisition of fast MREG data enable a robust separation of the cardiac and respiratory pulsations because of absence of signal aliasing over the very low frequencies (Kiviniemi et al., 2016; Huotari et al., 2019; Raitamaa et al., 2019).

During inspiration, the reduced intrathoracic pressure induces outflow of venous blood from brain that is counterbalanced by an inward movement of CSF, which is a necessary consequence of the confinement of brain within the incompressible dural venous sinuses and intracranial space (Klose et al., 2000; Yamada et al., 2013; Dreha-Kulaczewski et al., 2015; Vinje et al., 2019). This mechanism is bound also to change the perivenous CSF space, which, together with counterphase venous blood volume changes, creates a perivenous CSF pump. In addition, pulmonary ventilation and upper airway resistance increase during sleep (Wiegand et al., 1989; Trinder et al., 1992; Worsnop et al., 1998; Sowho et al., 2014). The increased intrathoracic ventilation pressures may consequently promote the (peri)venous CSF pumping action in the brain, manifesting in increased respiratory frequency power in the MREG signal (Dreha-Kulaczewski et al., 2015; Matsumae et al., 2019; Vinje et al., 2019). In addition, our results showing decreased spectral entropy in the respiratory frequency range during N1–2 sleep are in accord with findings that the respiratory pattern is more regular during NREM sleep compared with waking (Malik et al., 2012). The increased and stabilized physiological pulsations during sleep would likely increase CSF flow toward the neuropil, which might inflate the extracellular space, which has been shown to occur during sleep/wake transitions (Xie et al., 2013).

Furthermore, the increased respiratory and very low-frequency brain pulsations in NREM sleep overlap in posterior brain regions, which may be mediated by shared autonomic pressure control mechanisms (Özbay et al., 2019). In line with previous literature (Ma et al., 2016; Özbay et al., 2019), we suppose that the physiological pulsations may overpower neurovascularly coupled activity in the primary sensory processing areas during mainly N2 sleep. Thus, the enhanced brain clearance in light N2 sleep may arise in cortical areas that process sensory information in the awake state (Downar et al., 2000).

### Cardiovascular pulsations enhance in somatosensory cortex

Microscopic studies in rodent brain have shown that parenchymal cardiovascular pulsatility is the main contributor to CSF convection (Iliff et al., 2013; Mestre et al., 2018), whereas our whole brain analysis results do not show large-scale changes during NREM sleep. Cardiovascular pulsatility provides energy substrates supporting neuronal activity, which overall stays stable. However, we saw increased cardiac power in somatosensory cortex, a region that is known to remain active during sleep (Ramot et al., 2013). Lecci et al. (2017) found that a 0.02 Hz oscillation occurred in somatosensory cortex in NREM sleep in mice and humans and went on to suggest that such oscillations are mainly caused by sleep spindles tightly coupled with heart rate. Although the present data suggest that vasomotor and respiratory pulsations dominate throughout the neuropil during sleep, the interactions between the physiological pulsations need to be further investigated.

### Coupling between EEG and MREG pulsations

Recent studies have shown that the delta power changes in EEG are tightly related to BOLD activity changes along with faster CSF flow (Fultz et al., 2019), and that higher influx of CSF in the brain parenchyma allows increased solute transport from the brain (Hablitz et al., 2019). Fultz et al. (2019) showed that the onset of slow-delta EEG occurred 6.4 s before the CSF peak and concluded that such coupling was strongest during sleep. In addition, they found that BOLD and CSF signals were anticorrelated in the cortical gray matter, thus suggesting an alternation between BOLD and CSF flow, in accord with the constraint of constant intracranial volume. Interestingly, recent work showed that controlled deep inspirations during wakefulness were followed by fMRI and CSF changes similar to those occurring during NREM 1 and 2 sleep, thus suggesting that respiration is actually a driver for EEG and fMRI signal (Picchioni et al., 2021). Along the same lines, an increased respiratory effect on neuronal brain activity has been detected in intracranial needle measurements and MREG studies of epileptic patients (Zelano et al., 2016; Herrero et al., 2018; Kananen et al., 2018; 2020). Our findings suggest that the main changes in EEG occurred in slow oscillation power, which increased in concert with higher pulsation strength and reduced spectral entropy. Mechanistically, slow oscillation power increases along with elevated interstitial electrolyte levels and narrowing of the intercellular space (Xie et al., 2013; Ding et al., 2016; Hablitz et al., 2019); such mechanisms are likely to also occur during increased SWA human sleep. Because pulsation changes appeared in early sleep (NREM 1–2) and overlapped with EEG slow oscillation changes, we argue that these findings could reflect a direct physiological interaction between electrohydrodynamic pulsations rather than arising from an interaction with the faster neurophysiological pulsations that markedly decline in power during sleep.

### Limitations

The Awake scan was performed at 4:00–6:00 P.M. and the Sleep scan at 6:00–8:00 A.M., so circadian rhythms might have added a confound to our results. To minimize that possibility, we selected for analysis those segments that included the highest amount of sleep according to AASM sleep scoring criteria. In terms of naturalistic sleep, the fMRI laboratory is a challenging environment for attaining deep sleep; unfortunately, we only observed one epoch of N3 sleep. We conducted the sleep deprivation before the Sleep scan session to help subjects fall asleep in the scanner. Although somewhat effective, this intervention may have increased sympathetic activity, which is described to occur after sleep deprivation (Tobaldini et al., 2017). The sleep deprivation took place at home instead of the laboratory environment, so we are reliant on surrogate measures of sleep deprivation in some subjects.

We did not perform screening for sleep apnea or other sleep disorders as all included subjects were young and generally healthy. Our sample size was small (*n* = 12–15), calling for replication of some analysis in additional subjects. Subjective sleep quality was not assessed with validated questionnaires, which we consider to be another limitation. There was no a priori statistical power analysis. We had no criteria for the eating schedule for the subjects. Further work is required to investigate the exact source of different physiological changes over the whole 0.008–5 Hz frequency band that is accessible with direct current EEG and MREG.

### Conclusions

NREM sleep alters all three physiological brain pulsations, with the greatest power increases and spectral entropy decreases in the vasomotor and respiratory pulsations. In addition, we found a small power increase in cardiac frequency band. Electrophysiological slow oscillation power increased in brain regions overlapping with all pulsation changes, suggesting that these pulsations participate in sleep-related electrophysiological brain processes with an inverse relation to pulsation frequency. This inverse frequency change robustly reduces entropy of the brain physiological signal spectrum, enabling a clear dissociation of sleep and awake states from MREG data. We suggest the physiological brain pulsations alongside slow-wave electrophysiological changes in NREM sleep likely contribute to elevated CSF flow.
